# Single Nucleotide Polymorphism-Based Real-Time PCR Screening Assay for Rapid Tracking of Bacterial Infection Clusters To Complement Whole-Genome Sequencing Efforts during Outbreak Investigations

**DOI:** 10.1128/spectrum.03036-22

**Published:** 2022-10-17

**Authors:** Janina Treffon, Bianca Heppner, Julia Eismann, Julia Bothe, Birgit Omengo, Alexander Mellmann

**Affiliations:** a University Hospital Münster, Institute of Hygiene, Münster, Germany; b inno-train Diagnostik GmbH, Kronberg, Germany; Michigan State University

**Keywords:** cluster detection, genome sequencing, single nucleotide polymorphisms, signature search, real-time PCR, software evaluation

## Abstract

Infection clusters of multidrug-resistant bacteria increase mortality and entail expensive infection control measures. Whereas whole-genome sequencing (WGS) is the current gold standard to confirm infection clusters, PCR-based assays targeting cluster-specific signatures, such as single nucleotide polymorphisms (SNPs) derived from WGS data, are more suitable to initially screen for cluster isolates within large sample sizes. Here, we evaluated four software tools (SeqSphere^+^, RUCS, Gegenees, and Find Differential Primers) regarding their efficiency to find SNPs within WGS data sets that were specific for two bacterial monospecies infection clusters but were absent from a WGS reference data set comprising several hundred diverse genotypes of the same bacterial species. Cluster-specific SNPs were subsequently used to establish a probe-based real-time PCR screening assay for *in vitro* differentiation between cluster and noncluster isolates. SeqSphere^+^ and RUCS found 2 and 24 SNPs for clusters 1 and 14 and 24 SNPs for cluster 2, respectively. However, some signatures detected by RUCS were not cluster specific. Interestingly, all SNPs identified by SeqSphere^+^ were also detected by RUCS. In contrast, analyses with the remaining tools either resulted in no SNPs (with Find Differential Primers) or failed (Gegenees). Design of six cluster-specific real-time PCR assays enabled reliable cluster screening *in vitro*. Our evaluation revealed that SeqSphere^+^ and RUCS identified cluster-specific SNPs that could be used for large-scale screening in surveillance samples via real-time PCR, thereby complementing WGS efforts. This faster and simplified approach for the surveillance of bacterial clusters will improve infection control measures and will enhance protection of patients and physicians.

**IMPORTANCE** Infection clusters of multidrug-resistant bacteria threaten medical facilities worldwide and cause immense health care costs. In recent years, whole-genome sequencing (WGS) has been increasingly applied to detect and to further control bacterial clusters. However, as WGS is still expensive and time-consuming, its exclusive application for screening and confirmation of bacterial infection clusters contributes to high costs and enhanced turnaround times, which many hospitals cannot afford. Therefore, there is need for alternative methods that can enable further surveillance of bacterial clusters that are initially detected by WGS in a faster and more cost-efficient way. Here, we established a system based on real-time PCR that enables rapid large-scale sample screening for bacterial cluster isolates within 7 days after the initial detection of an infection cluster, thereby complementing WGS efforts. This faster and simplified surveillance of bacterial clusters will improve infection control measures and will enhance protection of patients and physicians.

## INTRODUCTION

The rising incidence of multidrug-resistant (MDR) bacteria in medical facilities represents a global problem (1, 2). In particular, the accumulation and spread of MDR bacteria in intensive care units is a trend of great concern, as it increases mortality rates of vulnerable patients and causes high financial expenses due to extensive infection control measures (3–6). Rapid detection and tracking, i.e., surveillance, of these bacterial clusters are therefore essential to improve patient safety and minimize financial costs.

Pathogen surveillance includes two steps: (i) sensitive screening assays that enable the identification of certain strains within hundreds of samples and (ii) specific confirmation assays that verify affiliation of these strains to, for example, particular clusters or groups. In recent years, whole-genome sequencing (WGS) has been increasingly applied to surveil drug-resistant pathogens (6, 7). Due to its high discriminatory power, this technique facilitates perfect confirmation of strain affiliation. However, as WGS is still time-consuming and expensive, it is not the best method for screening large sample sizes (7). For the first time during the large outbreak of enterohemorrhagic Escherichia coli O104:H4 in 2011, outbreak strain-specific PCRs targeting either unique genes or single nucleotide polymorphisms (SNPs) were used to screen for outbreak-associated isolates, which simplified and accelerated pathogen surveillance (8, 9). Later, a similar approach was developed for Mycobacterium tuberculosis, but with a rather long development time of more than a month (10–12). Usually, these PCR screening assays are established based on WGS data of a few putative cluster strains. Once developed, the PCR approaches can be applied as a good alternative to WGS for high-throughput screening of hundreds of samples, as PCR does not require an extensive laboratory infrastructure and allows rapid differentiation between cluster and sporadic isolates. Subsequently, only the cluster affiliation of selected strains identified in the screening assay has to be confirmed by WGS, which greatly reduces overall costs and turnaround times.

The crucial point for the successful development of cluster-specific PCR-based screening assays is the identification of signatures, i.e., SNPs or short unique sequences within the genome sequences of cluster-related isolates that define cluster-specific genotypes. For this, extensive bioinformatic analyses are necessary to determine appropriate signatures for primer and probe design. So far, many software tools have been developed that localize unique signatures in a specified target genome(s) (9, 13–21). However, these tools all differ in handling, turnaround times, and output, and a head-to-head evaluation to determine the specificity of the tools is still missing.

Here, we evaluated four software tools (SeqSphere^+^, RUCS, Gegenees, and Find Differential Primers) regarding their ability to find cluster-specific signatures using epidemiological and WGS data from a published outbreak cluster of the clinically relevant bacterial pathogen Acinetobacter baumannii (22). The most efficient software tools were used to develop a simple and rapid pipeline combining WGS and bioinformatic analysis to establish a real-time PCR-based screening assay for tracking of specific bacterial clusters that could be applied alternatively to screening exclusively done by WGS. Proof-of-principle testing was done by analyzing a second A. baumannii cluster and included the design and *in vitro* evaluation of cluster-specific SNP assays.

(Part of this work was presented at the 73rd Annual Meeting of the German Society for Hygiene and Microbiology e.V. in 2021 and the 2022 5th Munich Point-of-Care Testing Symposium: New Horizons for Cross-Sectional Technologies and Extended Application Areas.)

## RESULTS

### Identification and validation of cluster-specific signatures.

Prior to software evaluation, we tested the functionality of the four software tools SeqSphere^+^, RUCS, Gegenees, and Find Differential Primers by using a set of three A. baumannii whole-genome sequences (target genome, strain OC053; reference genomes, strains OC022 and OC077). All tools were functional, as they identified OC053-specific signatures or primers and probes that did not occur in strains OC022 and OC077 (see Table S1 in the supplemental material).

**FIG 1:**
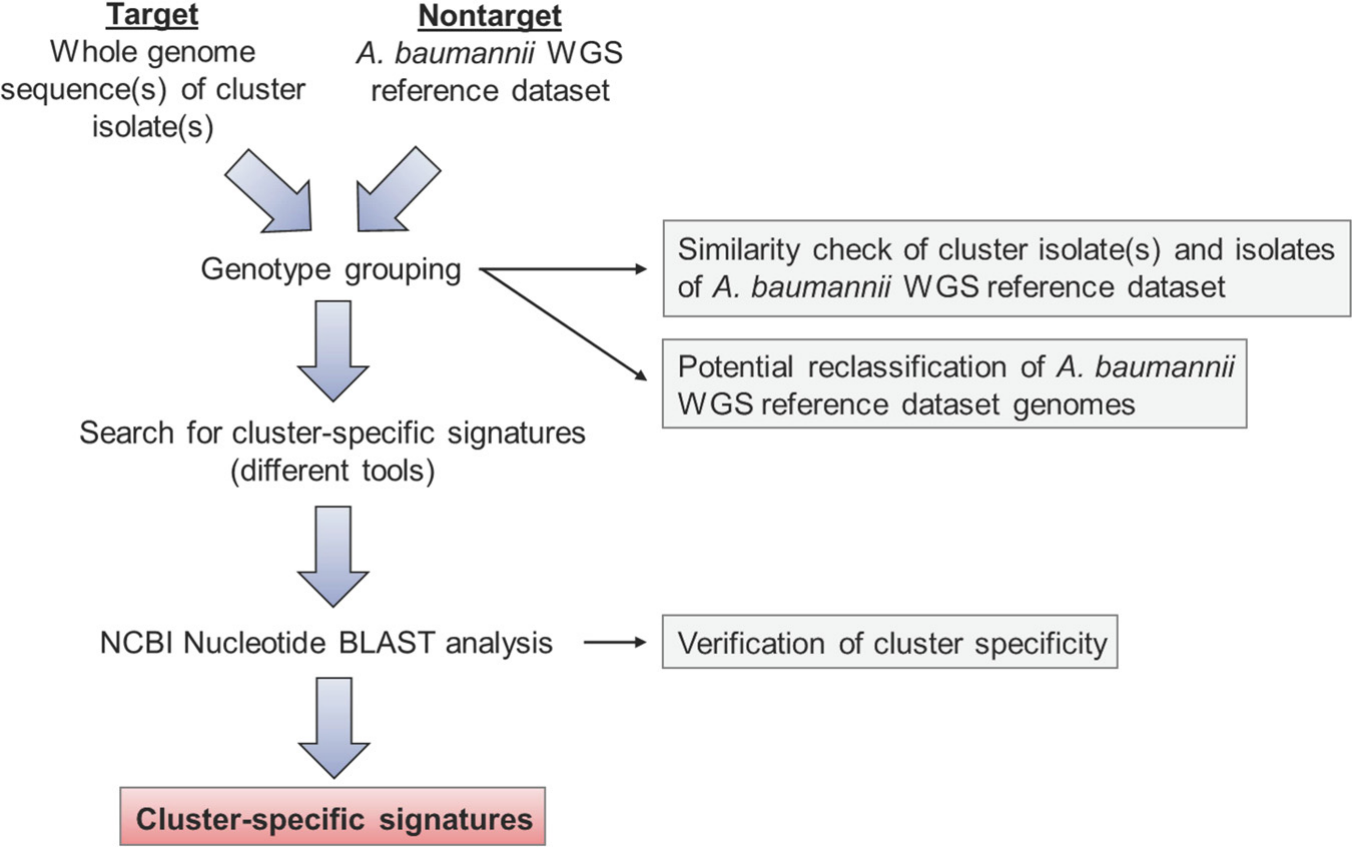
Workflow of bioinformatic analysis for identification of cluster-specific signatures.

Subsequently, the software evaluation was performed. To evaluate the potential of the software tools to identify cluster-specific signatures, we used WGS data of a published A. baumannii outbreak cluster (22), named cluster 1 (Table S2). The bioinformatic workflow is depicted in Fig. 1. Input data included the genome sequence of cluster index isolate OC019, which was used as target, and an A. baumannii WGS reference data set comprising 481 different A. baumannii genotypes (see Table S3). All 481 genomes were freely available at NCBI or the Institute of Hygiene, Münster. In pretests, we noticed that a high similarity between the target genome and any of the reference genomes caused the detection of signatures that were exclusively present in the analyzed target genome, i.e., the cluster index isolate, but were absent in the remaining strains of the cluster. As such strain-specific signatures are unsuitable to track whole bacterial clusters, prior to signature search we constructed minimum spanning tree (MST) based on the core genome multilocus sequence tag (cgMLST) allelic profiles of the target genome and A. baumannii WGS reference data set identified with SeqSphere^+^ to group similar genotypes (Fig. S1). In this MST, isolate OC020 of the A. baumannii WGS reference data set displayed an allelic distance of 1 to the cluster index isolate. We therefore moved the genome sequence of OC020 from the A. baumannii WGS reference data set to the target group for further analysis.

Next, we started the search for cluster-specific signatures. Analysis time and results varied considerably between the tools (Table 1). Investigations with Find Differential Primers took 30 h 22 min and did not yield any primers or probes. Analysis with Gegenees failed after approximately 5 days due to an assertion failure. In contrast, SeqSphere^+^ and RUCS identified 2 SNPs and 24 sequences within 40 min and 1 h, respectively.

**TABLE 1:** Identification of signatures specific for cluster 1 with SeqSphere^+^, RUCS, Gegenees, and Find Differential Primers

Tool	Results of signature analysis	Results of BLAST analysis
SeqSphere^+^	2 SNPs	2 SNPs do not occur in ACB complex^*a*^ genomes
RUCS	24 sequences	9 sequences contain SNPs that do not occur in ACB complex genomes
Gegenees	Analysis failed due to assertion failure	Not done
Find Differential Primers	0 primers and probes	Not done

a ACB complex = A. calcoaceticus-A. baumannii complex.

To verify the cluster specificities of the identified signatures, using NCBI Nucleotide BLAST we determined the absence of selected signatures in NCBI genomes of the Acinetobacter calcoaceticus-Acinetobacter baumannii (ACB) complex which, in addition to A. baumannii, included the species Acinetobacter calcoaceticus, Acinetobacter dijkshoorniae, Acinetobacter nosocomialis, Acinetobacter pittii, and Acinetobacter seifertii, which are phenotypically and genetically closely related to A. baumannii (23). In addition, this alignment analysis helped us to identify positions and bases of cluster-specific SNPs in sequences detected by RUCS, which lacked the function of indicating these SNPs in the output sequences.

BLAST analysis confirmed that both SNPs identified with SeqSphere^+^ were absent in genomes of the ACB complex, whereas only nine sequences detected with RUCS contained SNPs that did not occur in any ACB complex genome (Table 1).

### Screening of cluster-specific signatures via probe-based real-time PCR.

Real-time PCR allows fast and inexpensive high-throughput testing of hundreds of samples and is suitable for tracking bacterial clusters (8, 10). Therefore, we investigated whether signatures identified by SeqSphere^+^ and RUCS, which were not present in any genome of the ACB complex according to BLAST analysis, were suitable for cluster tracking by screening of cluster-specific signatures via probe-based real-time PCR. For this, we used a sample set of 54 isolates comprising all 15 cluster 1 isolates, 26 noncluster A. baumannii isolates, 5 ACB complex species, and 8 strains representing common hospital and environmental bacteria (Table S2).

Because detection of signatures, in our case SNPs, was done using only the index isolate of the cluster, it is possible that some of these SNPs were unique to the index isolate and did not occur in any other cluster strain. Furthermore, the A. baumannii WGS reference data set used during signature search and the BLAST database applied to verify cluster specificity of signatures comprise only a limited number of genomes. Thus, it might be that SNPs, which were classified as cluster specific (i.e., present in cluster isolates and absent in noncluster strains), still occurred in strains that were not part of the A. baumannii WGS reference data set or the BLAST database. To explore these potential limitations, prior to real-time PCR testing we investigated *in silico* SNP presence in all 15 cluster isolates and SNP absence in the other samples of the set. For this, we aligned SNPs that were not present in any genome of the ACB complex according to BLAST analysis (last column of Table 1) and their surrounding nucleotides against all 54 genomes of the sample set. This *in silico* analysis revealed that the investigated SNPs were cluster specific, as they did not occur in any noncluster isolate (Table S4). In fact, both SNPs detected by SeqSphere^+^ and two SNPs identified by RUCS were unique to the cluster. Interestingly, both tools found exactly the same two SNPs in the core genome of the cluster index isolate (Table S5). However, the remaining SNPs detected by RUCS were absent in some cluster isolates (Table S4).

For successful cluster screening, specific SNPs exclusively present in all cluster isolates are most suitable, while detection of SNPs that are absent in some cluster isolates could cause false-negative results. As SeqSphere^+^ and RUCS identified two SNPs that were exclusively present in all cluster 1 isolates (Table S4), we selected both SNPs (SNP1 and SNP2) for primer and probe design and tested their suitability for the detection of A. baumannii cluster 1 isolates by probe-based real-time PCR.

**FIG 2:**
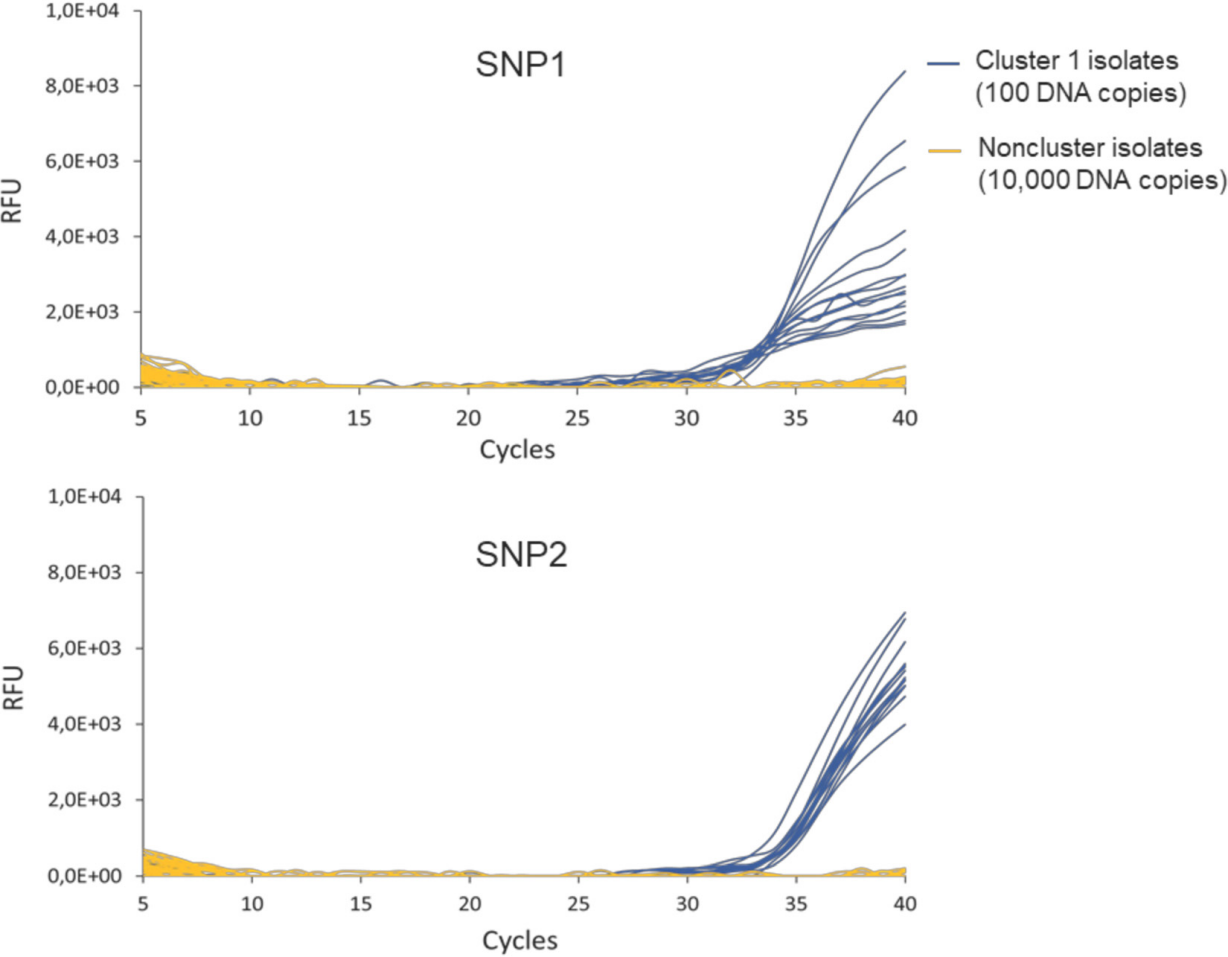
Probe-based real-time PCR detection of A. baumannii isolates of cluster 1, accomplished using primers and probes that targeted two SNPs identified by SeqSphere^+^ and RUCS: SNP1 and SNP2. Specificity was evaluated by monitoring real-time amplification of 100 copies of DNA extracted from 15 A. baumannii cluster isolates (OC015 to OC029) (dark blue), 10,000 copies of DNA isolated from 26 A. baumannii noncluster isolates (OC002, OC011, OC012, OC013, OC030, OC038, OC041, OC043, OC045, OC046, OC047, OC048, OC049, OC053, OC054, OC055, OC057, OC063, OC066, OC067, OC072, OC076, OC077, OC078, OC231, and ATCC 19606), five ACB complex species (A. calcoaceticus DSM 30006, *A. dijkshoorniae* LMG 29605, A. nosocomialis DSM 102856, A. pittii DSM 25618, and *A. seifertii* DSM 102854), and eight strains representing common hospital pathogens and environmental bacteria (C. difficile DSM 1296, E. cloacae DSM 30054, E. faecium DSM 20477, E. coli DSM 30083, K. pneumoniae DSM 30104, P. aeruginosa DSM 50071, S. aureus DSM 20231, and S. epidermidis DSM 20044) (orange).

As expected, primers and probes for detection of SNP1 and SNP2 successfully amplified DNA extracted from all 15 cluster 1 strains and failed to detect DNA of the remaining 39 noncluster strains (Fig. 2; Table S6). Therefore, these results reflected the cluster affiliation depicted in the MST based on WGS data (Fig. S2) and confirmed the preceding *in silico* analysis. Furthermore, the 16S rRNA gene was detectable in all samples, which demonstrated assay functionality (Table S7).

To show the ubiquitous applicability of our system, we looked for specific signatures in isolates of another A. baumannii cluster, cluster 2 (Table S2). For this, a search for cluster-specific signatures was performed only with the most efficient bioinformatic tools, SeqSphere^+^ and RUCS, as described above. We classified the cluster 2 index isolate OC031 and all strains of the A. baumannii WGS reference data set that formed a cluster with this isolate according to genotype grouping with MST (here, strain DB075) as targets and conducted the bioinformatic analysis including a signature search and BLAST alignment of the resulting signatures against genomes of the ACB complex.

Again, the tools identified signatures within an hour. While SeqSphere^+^ detected 14 SNPs, of which 13 did not occur in any ACB complex genome according to the NCBI BLAST alignment, RUCS found 24 sequences, of which 18 sequences contained SNPs that were absent in ACB complex genomes stored in the NCBI Nucleotide Collection database (Table 2). Again, the 13 SNPs identified by SeqSphere^+^ were also detected by RUCS. This time, the SNPs were located in either the core genome or the accessory genome of the cluster index isolate (Table S5).

**TABLE 2:** Identification of signatures specific for cluster 2 with SeqSphere^+^ and RUCS

Tool	Results of signature analysis	Results of BLAST analysis
SeqSphere^+^	14 SNPs	13 SNPs do not occur in ACB complex^*a*^ genomes
RUCS	24 sequences	18 sequences contain SNPs that do not occur in ACB complex genomes

a ACB complex = A. calcoaceticus-A. baumannii complex.

As before, SNPs that were absent in ACB complex genomes (last column of Table 2) were tested regarding their suitability for cluster tracking by screening of cluster-specific signatures via real-time PCR. This time, we applied a sample set of 30 isolates comprising DNAs of all 11 suspected cluster 2 isolates, 11 noncluster A. baumannii strains, 4 ACB complex species, and 4 common nosocomial and environmental bacteria (Table S2). Again, we aligned the SNPs and their surrounding nucleotides against all 30 genomes of the sample set to validate SNP presence in all 11 cluster 2 isolates and SNP absence in the other samples of the set *in silico*. The analysis revealed that the same three SNPs identified by SeqSphere^+^ and RUCS were absent in some cluster isolates (Table S8). All other SNPs were unique to the cluster and did not occur in any other sample of the set.

Of the SNPs that were uniquely present in the cluster 2 strains according to our *in silico* analysis (10 SNPs identified by SeqSphere^+^ and 15 SNPs found by RUCS) (Table S8), we randomly selected two SeqSphere^+^ SNPs (SNP3 and SNP4) and two RUCS SNPs (SNP5 and SNP6), designed SNP-specific primers and probes, and tested their abilities to amplify DNA of all 30 isolates of the sample set.

As expected, all primers and probes failed to amplify DNA from noncluster isolates (Table 3). Unexpectedly, only two primer and probe pairs (SNP5 and SNP6) were able to amplify DNA of all cluster isolates and thus allowed a cluster classification similar to that depicted in the MST based on WGS data (Fig. S2). Oligonucleotides for the detection of SNP3 and SNP4 failed to detect some cluster isolates (for SNP3, strains OC032, OC036, and OC044; for SNP4, strains OC032 and OC034). Nevertheless, the 16S rRNA gene was detectable in all samples, thereby demonstrating assay functionality (Table S7).

**TABLE 3:** Validation of primers and probes designed for detection of cluster 2 via probe-based real-time PCR^*a*^

Isolate category	Species	Strain	Copy no.	*C_T_* value			
				SNP3	SNP4	SNP5	SNP6
Cluster isolate	A. baumannii	OC031	100	37.14	36.28	36.52	32.40
Cluster isolate	A. baumannii	OC032	100	ND	ND	35.58	31.70
Cluster isolate	A. baumannii	OC033	100	37.01	35.78	35.96	30.63
Cluster isolate	A. baumannii	OC034	100	36.97	ND	35.55	30.56
Cluster isolate	A. baumannii	OC035	100	36.12	36.87	35.56	31.59
Cluster isolate	A. baumannii	OC036	100	ND	37.31	35.60	32.65
Cluster isolate	A. baumannii	OC037	100	36.73	37.17	35.95	32.44
Cluster isolate	A. baumannii	OC038	100	36.80	36.04	36.47	32.61
Cluster isolate	A. baumannii	OC039	100	37.50	36.24	37.58	31.25
Cluster isolate	A. baumannii	OC040	100	37.07	39.00	35.45	32.82
Cluster isolate	A. baumannii	OC044	100	ND	35.21	35.37	32.37
Noncluster isolate	A. baumannii	OC002	10,000	ND	ND	ND	ND
Noncluster isolate	A. baumannii	OC019	10,000	ND	ND	ND	ND
Noncluster isolate	A. baumannii	OC023	10,000	ND	ND	ND	ND
Noncluster isolate	A. baumannii	OC030	10,000	ND	ND	ND	ND
Noncluster isolate	A. baumannii	OC041	10,000	ND	ND	ND	ND
Noncluster isolate	A. baumannii	OC053	10,000	ND	ND	ND	ND
Noncluster isolate	A. baumannii	OC066	10,000	ND	ND	ND	ND
Noncluster isolate	A. baumannii	OC076	10,000	ND	ND	ND	ND
Noncluster isolate	A. baumannii	OC078	10,000	ND	ND	ND	ND
Noncluster isolate	A. baumannii	OC0231	10,000	ND	ND	ND	ND
Noncluster isolate	A. baumannii	ATCC 19606	10,000	ND	ND	ND	ND
Noncluster isolate	A. calcoaceticus	DSM 30006	10,000	ND	ND	ND	ND
Noncluster isolate	A. nosocomialis	DSM 102856	10,000	ND	ND	ND	ND
Noncluster isolate	A. pittii	DSM 25618	10,000	ND	ND	ND	ND
Noncluster isolate	*A. seifertii*	DSM 102854	10,000	ND	ND	ND	ND
Noncluster isolate	E. faecium	DSM 20477	10,000	ND	ND	ND	ND
Noncluster isolate	E. coli	DSM 30083	10,000	ND	ND	ND	ND
Noncluster isolate	P. aeruginosa	DSM 50071	10,000	ND	ND	ND	ND
Noncluster isolate	S. aureus	DSM 20231	10,000	ND	ND	ND	ND
No-template control		H_2_O		ND	ND	ND	ND

a Specificities of primers and probes were evaluated by monitoring real-time amplification of 100 copies of DNA extracted from 11 A. baumannii cluster isolates and 10,000 copies of DNA isolated from 11 A. baumannii noncluster isolates and 8 hospital-relevant, environmental, and skin bacteria. *C_T_*, threshold cycle; ND, no signal detection.

### Prospective application of the cluster typing system.

**FIG 3:**
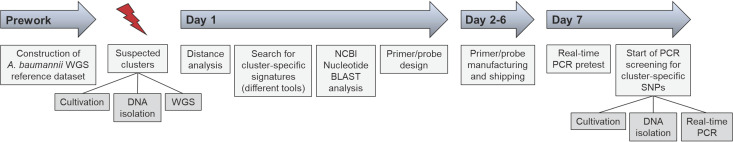
Workflow and turnaround time of bacterial cluster screening. Arrows indicate working days, and the work steps are shown below the arrows.

Bioinformatic analysis with SeqSphere^+^ and RUCS enabled identification of cluster-specific signatures that were detectable by real-time PCR and facilitated reliable differentiation between cluster and noncluster isolates. We suggest that our cluster screening system can be applied prospectively for tracking of suspected bacterial clusters. As fast delivery of results is essential during cluster tracking, we assessed the total turnaround time of our cluster typing system (Fig. 3). Upon detection of suspected bacterial clusters during clinical routine cultivation and WGS of one or more putative cluster isolates, the screening system can be executed. The system takes 7 days to complete the analysis, including the bioinformatic analysis and primer and probe design on day 1, manufacturing and shipping of oligonucleotides from days 2 to 6, and a real-time PCR pretest to assure functionality of primers and probes on day 7. Subsequently, large numbers of isolates can be screened by real-time PCR following sample collection, cultivation, and DNA isolation. This will complement WGS efforts and accelerate and simplify surveillance of bacterial clusters. Construction of the A. baumannii WGS reference data set was not included in our measurement, as such a reference data set can be prepared before the detection of suspected bacterial clusters. Similar to our methods here, the WGS reference data set should be constructed with freely available sequences from NCBI or other institutions to reduce application of expensive WGS.

## DISCUSSION

By performing a head-to-head evaluation of the four software tools, SeqSphere^+^, RUCS, Gegenees, and Find Differential Primers, with epidemiological and WGS data from two A. baumannii clusters, we identified efficient tools and established a simple workflow for the identification of cluster-specific SNPs. We demonstrated that these SNPs can be targeted by real-time PCR, which could facilitate fast and easy sample screening and could present an alternative to screening exclusively performed by WGS. Nevertheless, WGS is still required to identify a putative cluster isolate for screening assay development and to finally confirm cluster affiliations of selected strains.

SeqSphere^+^, RUCS, Gegenees, and Find Differential Primers were selected for evaluation because these tools have already been used successfully for detection of cluster-specific signatures (9, 13, 14, 22). In our analyses, we chose the cluster index isolates as targets and an A. baumannii WGS reference data set comprising several hundred diverse genotypes as nontarget to confine cluster-nonspecific SNPs. Genotype grouping prior to our search for cluster-specific signatures allowed us to identify strains of the A. baumannii WGS reference data set that were genetically similar to the cluster isolate. As classification of these strains as nontargets resulted in the identification of SNPs that were only present in the cluster index isolate and not detectable in the remaining cluster strains, we reclassified these strains as targets, which was essential to identify SNPs that were present in all cluster strains.

Analysis with Find Differential Primers and Gegenees delivered no signatures. In contrast, SeqSphere^+^ and RUCS were the most efficient tools, as they identified signatures in 40 min and 1 h, respectively. RUCS was most sensitive and identified more signatures than SeqSphere^+^. This was most likely due to the fact that RUCS looks for signatures in almost the complete genome (i.e., DNA sequences including coding and noncoding sequences common to all target samples), whereas SeqSphere^+^ only screens coding sequences to find SNPs. However, SeqSphere^+^ showed a high specificity and detected almost exclusively cluster-specific SNPs, while signatures identified by RUCS more often were cluster nonspecific. Nevertheless, to find and exclude signatures that were present in more than just the cluster genomes, outputs of both SeqSphere^+^ and RUCS had to be aligned against genomes of the ACB complex. Additionally, this alignment was essential to identify positions and bases of cluster-specific SNPs in signatures found by RUCS.

SNPs that were absent in the A. baumannii WGS reference data set and thus probably specific for the respective clusters were used to develop a probe-based real-time PCR assay for the detection of cluster-specific signatures. Our assay facilitated specific detection of cluster isolates and therefore allowed a cluster classification similar to that depicted in an MST based on WGS data (see Fig. S2 in the supplemental material). We decided to establish a probe-based real-time PCR assay, because it is very sensitive and robust. A more cost-efficient method that allows differentiation of cluster and noncluster isolates *in vitro* is high-resolution melting (HRM) analysis, which enables SNP discrimination by comparing melting temperatures of PCR products (24). HRM analysis requires only two primers and a DNA-binding fluorescent dye and therefore is easier and faster to establish than a probe-based real-time PCR. However, melting temperature depends on sequence length and percent GC content of the analyzed PCR product. Thus, HRM analysis cannot be used to differentiate between SNPs that do not change the amount of guanosine and cytosine in the target sequence, indicating that this method is more error-prone and less robust than probe-based real-time PCR.

Prior to real-time PCR testing, we aligned the SNPs and their neighboring nucleotides against genomes of the real-time PCR sample sets to verify SNP presence in all cluster isolates and SNP absence in all noncluster isolates. This *in silico* analysis revealed that all SNPs detected by SeqSphere^+^ and RUCS and controlled by BLAST alignment against genomes of the ACB complex were exclusively present in cluster isolates and absent in all noncluster isolates of the sample set. This indicated that construction of an A. baumannii WGS reference data set used during signature search, which has to be a compromise between representativeness of A. baumannii genotype diversity and a relatively small size to assure a rapid analysis, as well as BLAST alignment against genomes of the ACB complex are essential to facilitate the detection of cluster-specific SNPs.

Several studies have demonstrated that cluster isolates are heterogeneous and can differ by various SNPs from each other (22, 25). Nevertheless, our data revealed that applying only one isolate of each cluster as the target during the signature search could lead to the identification of SNPs that are common to all strains of the cluster and demonstrated the power of bioinformatic tools to work even in a worst-case scenario, when only limited WGS data are available. Admittedly, the tools also identified SNPs that were only present in a subset of cluster strains (Tables S4 and S8). To steer the signature search toward identification of SNPs that are common to all strains of a bacterial cluster, all available cluster isolates should be applied as targets during signature search.

Construction of a representative A. baumannii WGS reference data set as well as BLAST alignment against genomes of the ACB complex facilitated detection of cluster-specific SNPs. As we set up and tested the system retrospectively on well-characterized, sequenced bacterial strains, we were also able to investigate presence or absence of SNPs in the sample sets used for real-time PCR *in silico*. This additional control step enabled us to establish the assay with SNPs that were exclusively present in the cluster isolates and prevented false-negative or false-positive results, which can occur if SNPs that are absent in some cluster isolates or present in some noncluster isolates are used for cluster screening. However, if the system is applied prospectively, e.g., for tracking of suspected bacterial clusters, *in silico* investigation of SNPs cannot be performed, as sequencing data will be missing and development time is scarce. Therefore, to achieve correct isolate screening during source- and time-limited prospective tracking of bacterial clusters, we recommend targeting several SNPs instead of relying on just one SNP. Screening for several SNPs could also compensate loss of SNPs that are located on dynamic elements like plasmids or transposons, which might get lost during transmission events or laboratory cultivation. Furthermore, as design of SNP-specific primers and probes might not be successful the first time (see SNP3 and SNP4 data in Table 3), we suggest designing multiple primer and probe variants per SNP to save time during real-time PCR setup.

In our study, construction of the A. baumannii WGS reference data set, genotype grouping, and search for cluster-specific signatures were performed with the licensed software SeqSphere^+^. If a SeqSphere^+^ license is not available, free software tools like the SRA toolkit (https://github.com/ncbi/sra-tools) and WGS read-processing tools like Trimmomatic (26) and SKESA (27), as well as Parsnp (https://harvest.readthedocs.io/en/latest/content/parsnp.html) (28) and RUCS, can be applied for the construction of an A. baumannii WGS reference data set and subsequent bioinformatic analyses.

Four tools for extracting unique signatures from WGS data were evaluated in this study. There are also other tools, like KPATH, Insignia, TOPSI, kSNP3, ssGeneFinder, or PanSeq, available for performing, in principle, a similar job. However, those tools all suffer from certain limitations and therefore were not considered here. For example, due to a whole-genome multiple alignment strategy, KPATH is computationally extremely intensive and comes with a heavy scaling penalty when working with large genome data sets (18). By using a whole-genome pairwise alignment strategy, Insignia avoids the scaling problematic, but it does not offer an option for users to upload their own cluster sequences (19). TOPSI uses a BLAST-based, fast whole-genome progressive alignment strategy, but it relies on a cluster computing infrastructure with support for only a very specific job scheduler that makes installation difficult (20). kSNP3, an alignment- and reference-free SNP detection tool, is easy to install but requires various working steps and did not meet our “easy handling” criterion (16). In 2011, the software ssGeneFinder was successfully used to identify specific genetic targets for strains belonging to the E. coli O104:H4 outbreak that occurred in Germany (21). However, during our study the ssGeneFinder web server was not accessible. Finally, the PanSeq Novel Regions web service and the local version, which employs a MUMmer-based whole-genome progressive alignment strategy, were either not available during this study or produced no plausible results (15).

In conclusion, we determined that SeqSphere^+^ and RUCS are efficient software tools to identify cluster-specific signatures, provided that an appropriate A. baumannii WGS reference data set is constructed and that SNPs are validated via NCBI Nucleotide BLAST analysis. We demonstrated that these signatures can be targeted by real-time PCR to rapidly identify cluster relatedness of subcultured isolates among large sample sizes. This will complement WGS efforts and accelerate and simplify surveillance of bacterial clusters, thereby improving patient and physician safety. As Zhang et al. showed that real-time PCR can be successfully applied to enrichment cultures of stool samples (8), we assume that our assay also can be used to target outbreak strains in rudimentarily purified patient samples. However, this should be verified in future assays.

## MATERIALS AND METHODS

### Bioinformatic tools.

To find the most efficient tool for the detection of cluster-specific signatures, we evaluated performance and outcome of the stand-alone versions of the software tools SeqSphere^+^ version 8.3.1 (the “Find group-specific SNVs” function [SNVs are single nucleotide variants]) (17, 29), RUCS (the “Find unique core sequences” function; https://bitbucket.org/genomicepidemiology/rucs/src/master/) (13), Gegenees version 3.1 (https://www.gegenees.org/download/GegeneesHelp.html) (14), and Find Differential Primers version 0.1.4 (https://github.com/widdowquinn/find_differential_primers/tree/master) (9). All software tools were tested on an Intel Xeon Bold 6254 CPU with 3.10-GHz × 8 processor with 32 GB of RAM running with Ubuntu 18.04.5 LTS 64-bit. Memory usage of SeqSphere^+^ and Gegenees was scaled up to 21.3 GB and 25 GB, respectively, to allow better comparability. A detailed comparison of the software tools is given in Table S9.

### Functionality testing.

Prior to software evaluation, we tested functionality of the four software tools by applying the whole-genome sequences of three randomly selected A. baumannii strains, OC022, OC053, and OC077, which belonged to different MLSTs (Table S2). In each analysis, we selected strain OC053 as target and used the remaining two strains as reference genomes. Analysis settings were similar to that used during software evaluation, described below on.

### Construction of the A. baumannii WGS reference data set.

For construction of the A. baumannii WGS reference data set used to confine cluster-unspecific SNPs, we collected through 11 January 2021 a total of 1,022 A. baumannii genomes that were either downloaded from the NCBI Genome database (www.ncbi.nlm.nih.gov/genome/) or the NCBI Sequence Read Archive (https://www.ncbi.nlm.nih.gov/sra/) (30) or whole-genome sequences obtained at the Institute of Hygiene, Münster, Germany (Table S3). The final collection comprised 250 NCBI complete genomes and chromosomes, 633 genomes from 15 publications describing A. baumannii outbreaks and transmission events (22, 25, 31–43), and 139 genomes that originated from clinical A. baumannii isolates that were collected in different German hospitals. A. baumannii species level was confirmed by applying either Mash Screen analysis via the software SeqSphere^+^ (44) or the Species ID tool of PubMLST (https://pubmlst.org/bigsdb?db=pubmlst_rmlst_seqdef_kiosk) (45). Subsequently, all genomes were analyzed by (cgMLST) with SeqSphere^+^ (46). Applying the same software and the option “pairwise ignore missing values,” the cgMLST profiles were used to generate a (MST) in order to group similar genotypes. Based on data explored by Higgins and Prior et al., in our MST genomes with an allelic distance of ≤9 belonged to the same cluster (46). Forty-one genomes were excluded from MST due to low quality (Table S3), as we applied the “Exclude samples with missing values in more than 10% of distance columns” function, resulting in 981 genomes that formed 103 clusters. We kept all individual, nonclustering genomes and the central genome of each cluster and removed all 500 remaining genomes, resulting in an A. baumannii WGS reference data set containing 481 heterogeneous A. baumannii genotypes (Table S3). Further details are given in the supplemental methods.

### Detection of cluster-specific signatures.

We aimed to find specific signatures for two A. baumannii clusters that were detected at the University Hospital Münster, Germany. In principle, the different software tools should report genomic signatures (SNPs or short unique sequences) that are present only in the (index) isolate of the cluster, in comparison to—under ideal conditions—an infinitively large A. baumannii WGS reference data set comprising unrelated genotypes. In real life, this data set consisted of a representative number of A. baumannii genome sequences from public databases, which were collected and processed as described above to construct the A. baumannii WGS reference data set. This A. baumannii WGS reference data set and the index isolates of either cluster 1 or 2 were subjected to analysis with the bioinformatic tools SeqSphere^+^, RUCS, Gegenees, and Find Differential Primers. As the 1,022 A. baumannii genomes used for construction of the A. baumannii WGS reference data set also included genomes from local A. baumannii strains, we suspected a potential similarity between the index isolates of clusters 1 and 2 and genomes of the A. baumannii WGS reference data set, which could disturb the bioinformatic analysis. To verify this, prior to each analysis, we constructed an MST with SeqSphere^+^ based on the cgMLST allelic profiles of all input genomes, using the “Pairwise ignore missing values” function to group similar genotypes. Based on this MST, we subdivided the input genomes into targets and nontargets. While the cluster index isolate and all genomes of the A. baumannii WGS reference data set with an allelic distance of ≤9 were classified as targets, the remaining genomes of the A. baumannii WGS reference data set were classified as nontargets. We intended to find sequences between 300 and 600 nucleotides (nt) in length containing cluster-specific SNPs. If the tools identified sequences with a total length shorter than 300 nt, we manually added the flanking nucleotides using the software SnapGene Viewer 4.2.3 (https://www.snapgene.com/snapgene-viewer/) to provide templates of sufficient length for primer and probe design. Depending on the software tool, signatures were called only in coding regions, the core genome, or the complete genome (Table S9). Details about the bioinformatic tools and the analysis settings can be found in the supplemental methods.

### Alignment analysis with NCBI Nucleotide BLAST.

To verify the cluster specificity of the identified nucleotide sequences, we screened the sequences of each analysis for their absence in other Acinetobacter genomes via alignment analysis with the NCBI Standard Nucleotide BLAST web application (https://blast.ncbi.nlm.nih.gov/Blast.cgi?PAGE_TYPE=BlastSearch) (47, 48). In more detail, we performed a megablast analysis, looking for highly similar sequences between the cluster-specific signatures and genomes of the A. calcoaceticus-A. baumannii (ACB) complex (taxid 909768) stored in the NCBI Nucleotide Collection database to the exclusion of uncultured or environmental sample sequences. We performed BLAST analysis against genomes of the ACB complex, as they included the species A. calcoaceticus, *A. dijkshoorniae*, A. nosocomialis, A. pittii, and *A. seifertii*, which are phenotypically and genetically closely related to A. baumannii (23). Next to confirming cluster specificity of the identified nucleotide sequences, the alignment analysis with NCBI BLAST helped us to identify positions and bases of the cluster-specific SNPs in sequences identified by RUCS which, in contrast to SeqSphere^+^, lacked the function of indicating these SNPs in its output sequences.

Sequences of cluster isolates identified by SeqSphere^+^ or RUCS, which contained SNPs that did not occur in any other genome of the ACB complex stored in the NCBI Nucleotide Collection database, were aligned to the genomes of the sample sets for cluster screening by real-time PCR of either cluster 1 or 2. In detail, we used the “Align two or more sequences” function of NCBI Nucleotide BLAST (https://blast.ncbi.nlm.nih.gov/Blast.cgi) to assess presence of these SNPs in the respective cluster isolates and absence in all other genomes of the sample sets.

### Bacterial strains and cultivation.

In total, genomic DNA of (i) 51 A. baumannii strains (clinical isolates and type strain ATCC 19606), (ii) five ACB complex type strains (A. calcoaceticus DSM 30006, *A. dijkshoorniae* LMG 29605, A. nosocomialis DSM 102856, A. pittii DSM 25618, *A. seifertii* DSM 102854), and (iii) eight type strains representing common hospital pathogens and environmental bacteria (Clostridioides difficile DSM 1296, Enterobacter cloacae DSM 30054, Enterococcus faecium DSM 20477, Escherichia coli DSM 30083, Klebsiella pneumoniae DSM 30104, Pseudomonas aeruginosa DSM 50071, Staphylococcus aureus DSM 20231, and Staphylococcus epidermidis DSM 20044) was used to validate functionality of real-time PCR primers and probes. Twenty-six of 51 A. baumannii strains originated from two clusters that occurred at the University Hospital Münster, Germany. Cluster 1 consisted of 13 isolates that were collected during an outbreak from October 2013 to January 2014 as described by Willems et al. (22) and two subsequently identified strains. As index isolate P1a could not be recovered from glycerol stock, we excluded it from analysis and used the second isolate of the outbreak, isolate P2 (hereafter named OC019), as the index isolate. Cluster 2 included 11 isolates that were collected from October to December 2016. Here, isolate OC031 was the index isolate.

Bacteria were cultured on Columbia sheep blood agar (Oxoid Deutschland GmbH, Wesel, Germany) at species-specific temperatures (30 to 37°C). Prior to extraction of genomic DNA for real-time PCR analysis or WGS, bacteria were streaked on a fresh blood agar plate or cultivated in liquid lysogeny broth (LB) at 180 rpm. All bacterial strains analyzed by real-time PCR and their cluster affiliations are listed in Table S2.

Genotypes of all 51 A. baumannii strains were determined via MLST with the Pasteur (49) and Oxford schemes (50), using SeqSphere^+^ (Table S2). Similarity of genotypes was visualized by constructing an MST based on the allelic profile generated by cgMLST with SeqSphere^+^ (Fig. S2).

### Extraction of genomic DNA.

Genomic DNA analyzed by real-time PCR or WGS was extracted using the Monarch genomic DNA purification kit (New England Biolabs GmbH, Frankfurt am Main, Germany) for Gram-negative bacteria and the DNeasy blood and tissue kit (Qiagen, Hilden, Germany) for Gram-positive bacteria, following the instructions of the manufacturers. For isolation of staphylococcal DNA, 5 μL of a 6-mg/mL lysostaphin solution was added to the lysis buffer. DNA concentration was measured with a NanoDrop 2000c apparatus (Thermo Fisher Scientific, Wesel, Germany), Qubit 2.0 fluorometer (Thermo Fisher Scientific), or DS-11 FX fluorometer (Biozym, Hessisch Oldendorf, Germany), and DNA quality was checked by determination of the ratios of *A*_260_/*A*_280_ and *A*_260_/*A*_230_ values. DNAs with values of <1.4 were either treated according to the desalting and buffer exchange cleanup protocol from NEB or were extracted newly from the respective bacterial strain.

### Whole genome sequencing.

Bacterial genomic DNA was subjected to WGS on either a MiSeq platform (Illumina Inc., San Diego, CA, USA) or a Sequel II platform (Pacific Biosciences Inc., Menlo Park, CA, USA) at the Institute of Hygiene, Münster, Germany. WGS on the MiSeq platform and subsequent *de novo* assembly were performed as recently described (51). WGS on the Sequel II platform was conducted as follows: starting with approximately 1 μg of bacterial DNA, we constructed the sequence library using the SMRTbell Express template prep kit 2.0 (Pacific Biosciences Inc.) according to the manufacturer’s recommendations and subsequently loaded the library onto the Sequel II system for a 15-h data collection run. The resulting long-read sequencing data were then assembled, applying the microbial assembly pipeline within the SMRT Link software version 9 (Pacific Biosciences Inc.), using default parameters except for the genome size, which was adopted to the anticipated genome size of the sequenced species (e.g., 3.8 Mb for A. baumannii).

### Probe-based real-time PCR.

A real-time PCR with fluorophore- and quencher-labeled probes was established to detect A. baumannii cluster isolates. Cluster isolates were identified via cluster-specific SNPs that were targeted by primers to enable design of generic probes that would bind to conserved regions within the DNA sequences flanking cluster-specific SNPs. Primers contained the SNP base and a mismatch nucleotide to enhance amplification specificity (52) (Table S10, Fig. S3). Probes were labeled with the quencher Eclipse at the 3′ end and the fluorophore carboxyfluorescein at the 5′ end. For detection of cluster 1 isolates, two SNPs identified by both SeqSphere^+^ and RUCS (SNP1 and SNP2) were used. Isolates of cluster 2 were identified via two SeqSphere^+^ SNPs (SNP3 and SNP4) and two RUCS SNPs (SNP5 and SNP6). To control assay functionality, the 16S rRNA gene was targeted using generic oligonucleotides modified after the methods described by Nadkarni et al. (53) to amplify DNA of different bacterial species (Table S10). The 16S rRNA-specific probe was labeled with the Black Hole Quencher BHQ-2 at the 3′ end and the fluorophore cyanine-5 at the 5′ end. Design and manufacturing of primers and probes were performed by inno-train Diagnostik GmbH, Kronberg, Germany. Melting temperature, GC content, and lengths of primers and probes were determined with the IDT OligoAnalyzer tool (https://eu.idtdna.com/pages/tools/oligoanalyzer?returnurl=%2Fcalc%2Fanalyzer). The Multiple Primer Analyzer from ThermoFisher Scientific was used to detect potential formation of homo- or heterodimers (https://www.thermofisher.com/de/de/home/brands/thermo-scientific/molecular-biology/molecular-biology-learning-center/molecular-biology-resource-library/thermo-scientific-web-tools/multiple-primer-analyzer.html).

Functionality of cluster-specific primers and probes was evaluated by monitoring real-time amplification of 100 copies of DNA (0.375 pg) extracted from A. baumannii cluster isolates and 10,000 copies of DNA (37.5 pg) isolated from A. baumannii noncluster isolates and other hospital-relevant and environmental bacteria, assuming that one copy of bacterial DNA corresponded to 3.75 fg DNA (inno-train Diagnostik GmbH, written communication). We applied smaller amounts of DNA extracted from cluster isolates than of DNA isolated from noncluster strains to demonstrate primer and probe functionality even in a worst-case scenario, in which plenty DNA of noncluster isolates, but only limited DNA of cluster strains, would be available.

Real-time PCR was performed on a CFX96 C1000 Touch thermal cycler (Bio-Rad Laboratories GmbH, Feldkirchen, Germany) running with the Bio-Rad CFX Maestro software 1.1, applying the following thermal cycling conditions: 95°C for 2 min, 40 cycles including 95°C for 15 s and 60°C for 1 min (signal detection), and finally 20°C for 3 min. Lid temperature and ramp rate were set to 100°C and 5.0°C/s, respectively. Each 15-μL reaction contained 2× FluoMix, 900 nM forward and reverse primer, and 200 nM probe (all substances provided by inno-train Diagnostik GmbH). Evaluation of real-time PCR was done with the Bio-Rad CFX Maestro software, applying *Cq* determination mode regression to cycles 5 to 40.

### Data availability.

All Illumina raw reads generated in this study were submitted to the European Nucleotide Archive (http://www.ebi.ac.uk/ena/) under Project ID PRJEB48719. All Pacific Biosciences sequences generated in this study were submitted to NCBI under BioProject ID PRJNA779872. All other data supporting the findings of this study are available within the paper or in the supplemental material.
